# Syphilis at the Crossroad of Phylogenetics and Paleopathology

**DOI:** 10.1371/journal.pntd.0000575

**Published:** 2010-01-05

**Authors:** Fernando Lucas de Melo, Joana Carvalho Moreira de Mello, Ana Maria Fraga, Kelly Nunes, Sabine Eggers

**Affiliations:** 1 Departamento de Microbiologia, Instituto de Ciências Biomédicas, Universidade de São Paulo, São Paulo, Brazil; 2 Departamento de Genética e Biologia Evolutiva, Instituto de Biociências, Universidade de São Paulo, São Paulo, Brazil; University of Washington, United States of America

## Abstract

The origin of syphilis is still controversial. Different research avenues explore its fascinating history. Here we employed a new integrative approach, where paleopathology and molecular analyses are combined. As an exercise to test the validity of this approach we examined different hypotheses on the origin of syphilis and other human diseases caused by treponemes (treponematoses). Initially, we constructed a worldwide map containing all accessible reports on palaeopathological evidences of treponematoses before Columbus's return to Europe. Then, we selected the oldest ones to calibrate the time of the most recent common ancestor of *Treponema pallidum* subsp. *pallidum*, *T. pallidum* subsp. *endemicum* and *T. pallidum* subsp. *pertenue* in phylogenetic analyses with 21 genetic regions of different *T. pallidum* strains previously reported. Finally, we estimated the treponemes' evolutionary rate to test three scenarios: A) if treponematoses accompanied human evolution since *Homo erectus*; B) if venereal syphilis arose very recently from less virulent strains caught in the New World about 500 years ago, and C) if it emerged in the Americas between 16,500 and 5,000 years ago. Two of the resulting evolutionary rates were unlikely and do not explain the existent osseous evidence. Thus, treponematoses, as we know them today, did not emerge with *H. erectus*, nor did venereal syphilis appear only five centuries ago. However, considering 16,500 years before present (yBP) as the time of the first colonization of the Americas, and approximately 5,000 yBP as the oldest probable evidence of venereal syphilis in the world, we could not entirely reject hypothesis C. We confirm that syphilis seems to have emerged in this time span, since the resulting evolutionary rate is compatible with those observed in other bacteria. In contrast, if the claims of precolumbian venereal syphilis outside the Americas are taken into account, the place of origin remains unsolved. Finally, the endeavor of joining paleopathology and phylogenetics proved to be a fruitful and promising approach for the study of infectious diseases.

## Author Summary

Syphilis is a reemerging disease burden. Although it has been studied for five centuries, its origin and spread is still controversial. Did it accompany the evolution of the genus *Homo* and does it date back to more than a million years or did it emerge only after Columbus's return to Europe? Initially, to test the validity of a new interdisciplinary approach we constructed a worldwide map showing precolumbian human skeletons with lesions of syphilis and other related diseases (also caused by different treponemes). Then, we selected the oldest cases to estimate the timing of the treponemes' history, using their DNA sequences and computer simulations. This resulted in treponeme evolutionary rates, and in temporal intervals during which these microorganisms could have emerged. Based on comparisons with other bacteria, we concluded that treponematoses did not emerge before our own species originated and that syphilis did not start affecting mankind only from 1492 onwards. Instead, it seems to have emerged in the time span between 16,500 and 5,000 years ago. Where syphilis emerged, however, remains unsolved. Finally, the endeavor of joining as distinct fields as paleopathology and molecular biology proved to be fruitful and promising to advance our understanding of the rise and fall of the infectious diseases that have afflicted humans across time and space.

## Introduction

### Syphilis today

The widespread availability of effective antimicrobial therapy, in association with population screening, resulted in a considerable decline in the frequency of syphilis in the middle of the last century [1]. The mass campaigns around 1960 treated 46 out of 152 million people screened and, as a result, endemic treponematoses (bejel, pinta and yaws) were eradicated in many regions of the world. However, reservoirs persisted and expanded into poor communities with deficient hygiene and health care [2]. Recently, outbreaks of syphilis occurred in different subpopulations, also in association with AIDS. One decade old worldwide estimates report that there are 12 million new cases of syphilis per year [3], whereas the total number of people affected with the non-venereal treponemal diseases reached 2,5 million [4]. Due to venereal syphilis, 157,000 deaths, 460,000 abortions or stillbirths, 270,000 low-birth-weight babies and 270,000 cases of congenital syphilis were registered solely in Africa in 2002 [5]. The complete genome sequence of *Treponema pallidum*, the advancement of PCR technology, and the use of penicillin, were thought to make possible the eradication of venereal syphilis [6]. Nevertheless, syphilis and the endemic treponematoses are still a heavy burden today, but WHO's new goal to eradicate yaws by 2012 will hopefully change this situation [7]. This also means that the spread, virulence as well as the origin of syphilis needs to be further investigated.

### Historical background

Eros and Thanatos are linked not only in mythology, but also in the study of sexually transmissible diseases. The achievements of the sexual revolution (Eros) have been undermined by the fear of AIDS (Thanatos). After initially blaming homosexuals and Africans, research on the origin of AIDS is more objective today, facilitating treatment and quality of life. Similarly, during the last centuries, this phenomenon transformed the life threatening and shameful syphilis in an easily treatable disease. Artistic representations of syphilis clearly reflect this transition [8]. Shortly after the XVI^th^ century explosion of a sexually transmitted and disfiguring disease in Europe, Venus is shown as a source of contamination. Thereafter, women continue to be blamed for syphilis, in a satirical as well as stigmatizing way. More recently, not only artists, but also physicians and other researcher were inspired by the origin of syphilis. And for a long time not only women but also “savage” people discovered in the New World were blamed for syphilis. In contrast to the huge efforts to treat AIDS, syphilis and the other treponematoses are neglected diseases today, with a still mysterious origin.

### Syphilis and the other treponemal diseases

Treponemal diseases are caused by bacteria from the genus *Treponema*. Currently it is believed that different *Treponema* cause different diseases: *T. carateum* is responsible for pinta, *T. pallidum* subsp. *endemicum* leads to bejel (non-venereal syphilis or endemic syphilis), *T. pallidum* subsp. *pertenue* causes yaws, whereas *T. pallidum* subsp. *pallidum* is responsible for venereal and congenital syphilis (as reviewed in [9],[10]).

These diseases show overlapping clinical manifestations and tend to occur in distinct geographical settings, but today they all affect mainly people from poor communities with deficient health services and poor hygiene conditions. Syphilis is the only venereal treponemal disease, whereas pinta, bejel, and yaws are transmitted through skin and/or oral contact. A brief description of each of the four treponematoses follows (as reviewed in [9]–[11]).

Pinta is the only treponemal disease which does not affect skeletal tissues. Therefore it will not be further mentioned, since our purpose here is to use osteological data to estimate, along with a phylogenetic analysis, the origin of treponematoses.

**Figure 1:**
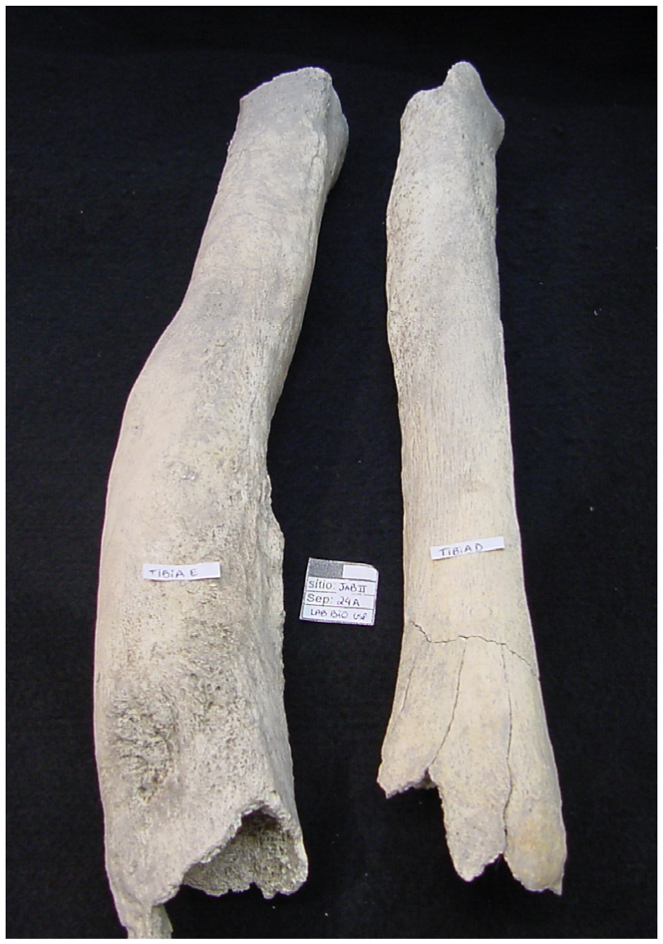
Tibiae with treponematosis. Note the anterior thickening on the tibia depicted on the left side. These bones were excavated at the Brazilian shell mound Jabuticabeira II, which dates to approximately 3,000 yBP.

Yaws occurs in hot regions with high humidity. In the late stages it is very destructive on bone (Figure 1), skin and mucous tissues. The most distinctive alteration in bone is the “saber shin”, where the tibia develops an abnormal buildup of bone particularly on the anterior and medial surface of the tibia.

Bejel occurs in dry, hot and temperate climates. Inflammations and destructive changes of tissues, including bone, occur only in the advanced stages of the disease. The clinical manifestations are intermediary between yaws and venereal syphilis.

Venereal syphilis is the most destructive of the treponemal diseases and today shows a worldwide distribution. Syphilis can result in painful osseous alterations, leading to “caries sicca” of the skull and also to “saber shin” tibiae. This disease is the only treponematosis that is able to affect inner organs, causing nervous system damage and cardiovascular complications. Venereal syphilis, much more than other treponematoses, can get through the placenta, causing congenital syphilis. Short and narrow central incisors (Hutchinson's incisors), small and dome shaped first molars (Moon molars), periostitis on long bones, “saber shin” tibiae and saddle shaped noses are the most important signs of congenital syphilis in the juvenile skeleton.

### Hypotheses on the origin of syphilis

One of the most popular hypotheses on the origin of syphilis states that it came from the Americas and was spread to Europe by Columbus' seamen [12],[13]. This is the Columbian hypothesis, which gained support through old ethnographic reports on treatment of syphilis with native plants in the New World [9].

Other hypotheses have also been proposed to explain the evolutionary history of the four treponemal diseases.

The Pre-Columbian hypothesis argues that syphilis and the other treponemal diseases were present throughout the New as well as the Old World in pre-Columbian times, but was misdiagnosed as leprosy in Europe [14]. Hackett, a convinced pre-Columbianist, proposed a scheme of mutational development linking the four treponemal diseases in 1963 [14]. Accordingly, a) pinta arose somewhere in Africa or Asia from an animal infection about 17,000 years before present (yBP) and spread to the rest of the world; b) mutations on the pinta causing microbe lead to yaws about 12,000 yBP, and spread to the world except to the Americas; c) bejel arose from yaws about 9,000 yBP in arid climates; and finally d) mutations on the bejel causing treponeme originated venereal syphilis about 5,000 yBP in south-west Asia and then spread to Europe and the rest of the world.

Finally, as discussed by some paleopathologists, the Unitarian hypothesis, states that treponematoses always had a worldwide distribution, where every social group had the kind of treponematosis appropriate to its geographic and climatic conditions and its stage of cultural development. Thus, according to this hypothesis, yaws, bejel, pinta and venereal treponematosis (or syphilis) are seen as adaptive responses of *Treponema pallidum* to peculiarities of environment, culture, and contact with other populations [14].

New data, instead of clarifying matters, contribute to an even more intricate scenario. For instance, osseous evidences of syphilis on pre-Columbian individuals from Europe [15]–[17], point against a New World origin of syphilis, while molecular data contradict the Unitarian hypothesis [18]. Consequently, although the issue has been discussed for five centuries, the origin of syphilis is not yet clear. Why? Do we have enough data to answer this question? Are there novel approaches to gain a better understanding of the when, how and where syphilis began troubling humans?

Recently, the study of the origin of syphilis has gained notoriety, when Harper et al. (2008) employed molecular genetics to analyze 21 genetic regions of *Treponema* stemming from geographically distinct areas [19]. According to the authors, “*T. pallidum arose in the Old World, in the form of non-venereal infection, before spreading with humans to the Middle East/Eastern Europe, in the form of endemic syphilis, and then to the Americas, in the form of New World yaws*”. Then, “*a T. pallidum strain from the Americas was introduced back into the Old World, probably as a result of the European exploration of the Americas, becoming the progenitor of modern syphilis-causing strains*” [19].

However, this work resulted in controversy, particularly due to the restricted number of DNA polymorphisms (SNPs) analyzed in two samples collected in a single location [20]. The approach used herein differs from that employed by Harper et al. (2008) [19]. Albeit it is based on the same genetic sequences, here we combine a Bayesian inference approach with time calibration points obtained from treponemal diseases in ancient human bones, resulting in confidence intervals for the likeliness of the origin of this human-pathogen interaction.

## Materials and Methods

### Tools to study origin and spread of diseases

Paleopathology studies origin and distribution of diseases based on historical accounts, iconography, ethnography, and ancient human remains. The most reliable source of information on health and disease of past populations are the lesions certain diseases, such as treponematoses, leave on ancient human skeletons [21]. Until recently, the great majority of studies on treponematoses were carried out on North American osteological collections [11]. However, the last years have seen a growing contribution of research on material stemming from other regions of the world [11], [16], [22]–[26]. Thus, exploring paleopathology to aid unraveling the origin of syphilis is mandatory.

A totally different approach refers to molecular biology and phylogenetics. The advances and joint study of molecular biology and phylogenetics allow the development of different analytical approaches which permit potentially more secure inferences based on genetic data. Recently this approach was used to a) unravel the degree of admixture of Neanderthal and modern man [27], b) to molecularly confirm cases of tuberculosis in ancient human remains [28], and c) to pinpoint treponemal diseases in mummies [29]. As more and more genomes of disease causing microorganisms are sequenced and published, more strength the molecular approach gains in expanding our knowledge on the history of the interaction between us and other species. Therefore, studying the origin of infectious disease using phylogenetics is indeed promising.

### Rationale of the present study

The integration of paleopathology and molecular biology harbors not only the advantages of each of these approaches alone, but enhances analytical quality, since it allows studying phylogenetics with the knowledge of the when and where certain bony lesions appeared first in the history of our ancestors. Syphilis and the other treponematoses represent natural candidates to test this approach.

Relying on the construction of a worldwide map containing all accessible reports on palaeopathological evidences of treponematoses before Columbus' return to Europe, we first explored the osteological data alone. Then we used some of the oldest dates of treponemal infection found in different regions of the globe to calibrate the time of the most recent common ancestor (TMRCA) of *Treponema pallidum* subsp. *pallidum*, *T. pallidum* subsp. *endemicum* and *T. pallidum* subsp. *pertenue*, using previously published sequences [19].

**Figure 2:**
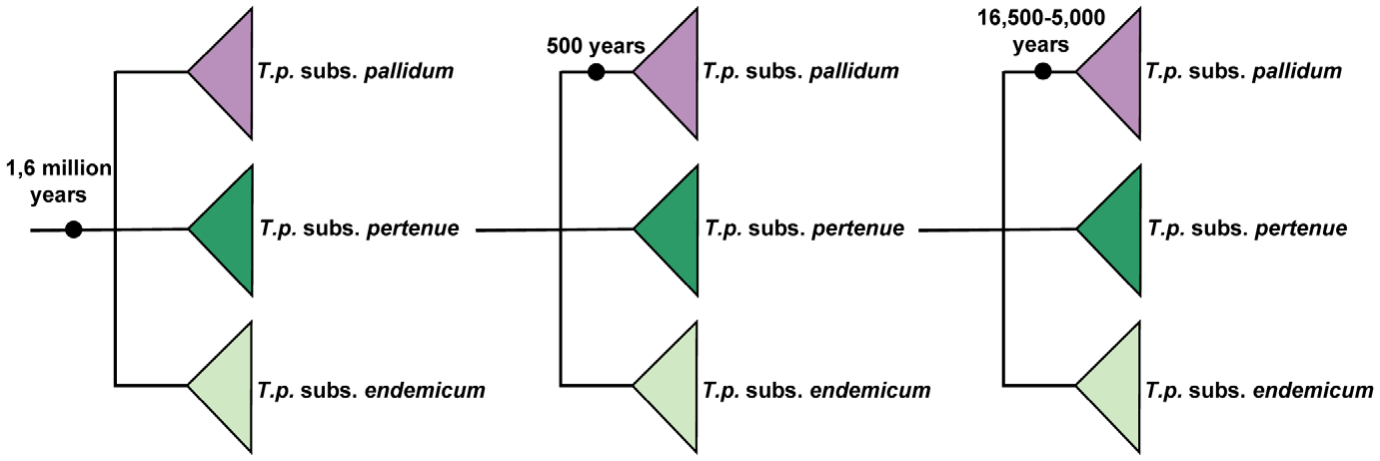
Hypotheses tested. The calibration points (obtained from the palaeopathological record) were used as node constrains for phylogenetic analyses to test the following hypotheses on the origin of treponematoses: A) *T. pallidum* emerged before modern humans evolved, using treponemal evidences in *Homo erectus* 1.6 million yBP. B) *T. pallidum* subsp. *pallidum* emerged from less virulent strains in Europe after Columbus' conquest 500 yBP. C) *T. pallidum* subsp. *pallidum* emerged in the time range between 16,500 yBP and 5,000 yBP.

More specifically, we explored three different scenarios where this integrative approach is investigated (Figure 2). First a) we tested if *T. pallidum* emerged before modern humans evolved, using treponemal evidence in *Homo erectus* [30]. Therefore we set a minimum bound of 1.6 million yBP on the root of the *T. pallidum* group. Furthermore, b) we explored the possibility that *T. pallidum* subsp. *pallidum* emerged from less virulent strains in Europe after Columbus' conquest, as recently suggested [19], setting the TMRCA of *T. pallidum* subsp. *pallidum* to 450–550 yBP. Finally, c) we analyzed if *T. pallidum* subsp. *pallidum* emerged in the time range between 16,500 yBP and 5,000 yBP. These dates refer to the entrance of the first humans into the New World [31], and to the most ancient probable osseous evidence of syphilis [32], respectively.

### Paleopathology

**Figure 3:**
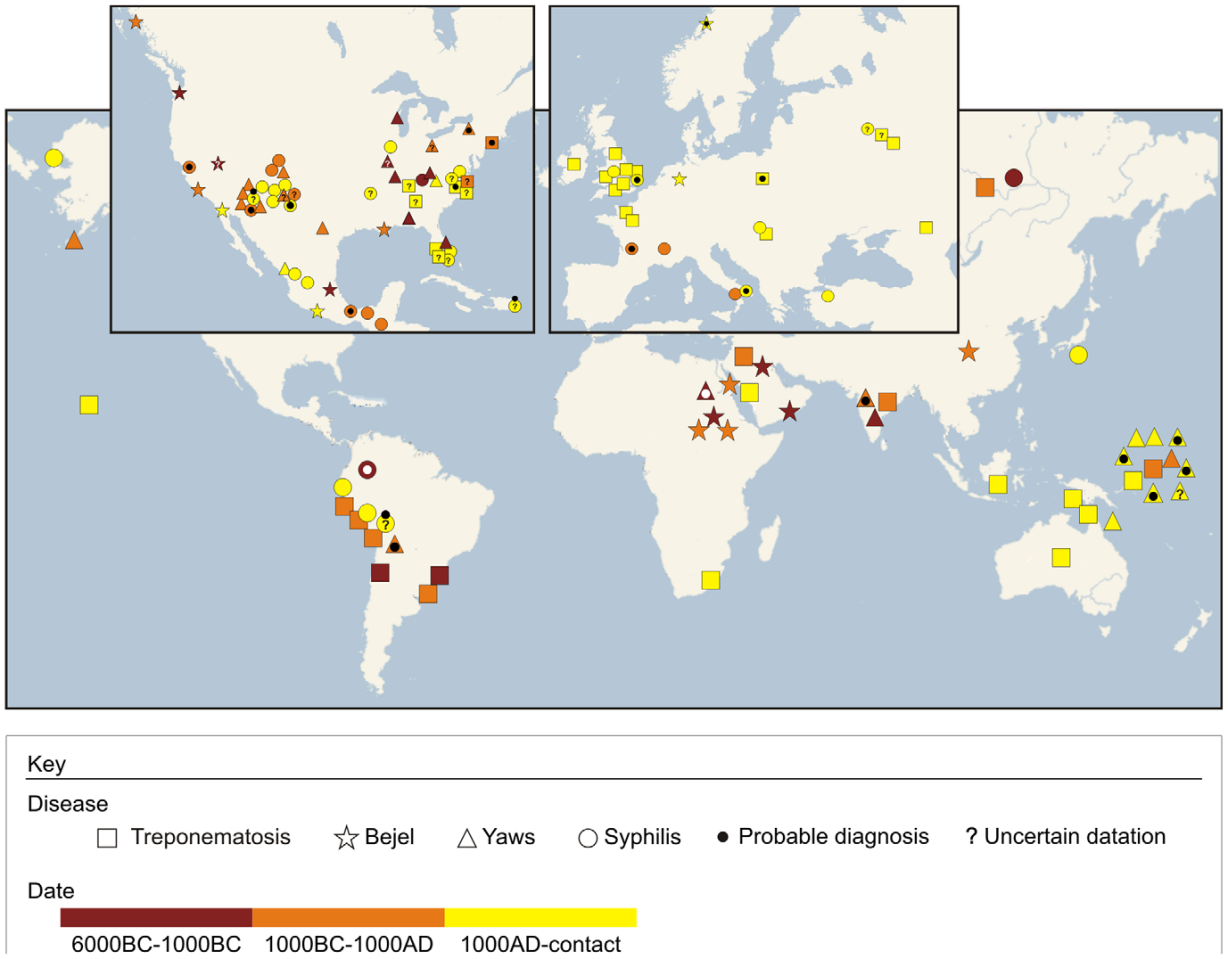
Pre-Columbian Treponematoses map. Temporal and geographic distribution of osseous evidences of different types of treponematoses. For the underlying bibliography, refer to Table S1.

The sources used to create the worldwide treponematoses map include primary publications (and the references within) on osteological evidences of treponematoses prior to the European conquest of the New World. These sources included: a) online published reports, b) traditional journals of free access through the University of São Paulo, c) books from the library of the Laboratório de Antropologia Biológica IBUSP, and d) reprints and pdf files requested from authors. The reported diagnoses of treponemal diseases were included in the table and map as published in the peer reviewed articles (Figure 3, Table S1). Wherever cited in the original reports, a specific diagnosis is presented in our map, such as (venereal) syphilis, congenital syphilis, bejel and yaws. However, in many cases, the authors did not distinguish between the different treponematoses and thus this broader term was used. All sources were divided into three main periods according to the intervals reported in the book The Myth of Syphilis [11]. In case a diagnosis or date of a certain treponematosis case was inconclusive, this was remarked (see legend of Figure 3, and Table S1). We used the nearest location possible to plot the sites on Google Maps. Special attention was given not to duplicate points reported more than once.

Statistical analyses were carried out to test temporal and geographic distribution of pre-Columbian cases of venereal syphilis, bejel and yaws. Cases diagnosed solely as treponematoses, and those reported in the original article with either uncertain age and/or diagnosis were excluded from the statistical analyses. The tests employed were Chi square and Fisher exact test, considering p<0,05 as statistically significant. Only significant p values are cited in the text.

### Phylogenetic analyses

Sequences from 20 *Treponema pallidum* isolates (9 *T. pallidum* subsp. *pallidum*, 9 *T. pallidum* subsp. *pertenue* and 2 *T. pallidum* subsp. *endemicum*) were obtained from previously published data [19], consisting of 21 genes and/or intergenic regions. GenBank accession numbers for all sequences used in this study are listed in Table S2. Some regions were not included in the analysis, since they could be involved in recombination events and/or present an elevated number of non-synonymous substitutions [19]. Sequence alignments were manually constructed and the final data set consisted of 5,412 base pairs (bp) of concatenated sequences. Since interspecific variation could lead to considerable overestimation of the divergence times for intraspecific data [33], no outgroup was included in the analyses. Thus, the approach used in this part of the study aims at explaining the existing genetic variation (and comparing the resulting rates of evolution with other bacteria) rather than inferring the order of evolutionary events.

Since *T. pallidum* is essentially a clonal population and there is no evidence of recombination in the database used in the present study, it is possible to estimate divergence times using a coalescent approach with appropriated calibration points. To avoid using a molecular clock rate estimated for bacteria not related to *T. pallidum*, such as *E. coli* [34], we used the palaeopathological record to calibrate the molecular clock. From the 128 osteological cases employed for the paleopathological analysis, we used six for the phylogenetic analyses. They refer to the oldest treponematosis case registered in the genus *Homo* and to the reports on the oldest cases of probable bejel, venereal syphilis and congenital syphilis in different parts of the World (see below). These records provided minimum and/or maximum age estimates to constrain nodes in the trees, as suggested by Achtman (2008) [35] and shown for our database in Figure 2.

Therefore, the different hypotheses on the origin of *T. pallidum* subspecies were tested using the Bayesian phylogenetic software BEAST V1.4.7. Given that evolutionary rates can vary between different organisms, we employed a coalescent model with constant population size under a relaxed molecular clock, which accounts for rate variation among tree branches [36]. Posterior distributions of parameters were obtained by two independent Markov Chain Monte Carlo (MCMC) analyses (chain length of 10 million, sampling every 1000 chains). The chains were compared to ensure convergence and merged to obtain final results.

For each hypothesis tested, we estimated the divergence times and substitution rates of the whole group of treponemes and of *T. pallidum* subsp. *pallidum*. To warrant a more conservative approach, we assumed either the lower (most recent/slower) or the upper (most ancient/faster) high posterior density (HPD) value, depending on the context.

Finally, we also tested hypotheses on the origins of *T. pallidum* subsp. *pallidum* using published data from whole genome comparison of the two *T. pallidum* subsp. *pallidum* strains available: *T. pallidum* subsp. *pallidum* S. Nichols [37] and *T. pallidum* subsp. *pallidum* SS14 [38]. Comparative genome analysis of these two strains performed by Matejkova et al. (2008) revealed a total of 327 SNPs within the 1.1 mega base genome [38]. Since genetic divergence (*K*) between two sequences is calculated as the number of substitutions per base pair, the *K* between these two strains is 2.96×10^−4^ substitutions per base pair (327/1.1×10^6^ base pairs). Assuming that the evolutionary rate does not vary over time, the genetic divergence can be used to calculate the time since their most recent common ancestor (TMRCA) using the following equation:
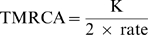
When the divergence time is known, the equation above can also be used to estimate the substitution rate per site per year. Therefore, we used two different divergence times to estimate rates of evolution in *T. pallidum* subsp. *pallidum*: *(i)* 500 yBP, as proposed by Harper et al. (2008) and others as described above [19], and *(ii)* the most ancient osseous evidences of syphilis, as for example the case from Colombia dated to circa 5,000 yBP [39] (Figure 3 and Table S1).

## Results/Discussion

Hominids and microbes have interacted ever since their first appearance in evolution. Africa, the cradle of hominids and humans, is well known as the home of a number of zoonotic diseases [40]. Although there are numerous genetic diseases with higher frequencies than expected in regions of endemic infectious diseases (such as sickle cell anemia in regions of endemic malaria), strikingly, no mutations have yet been reported that could help humans to overcome non-zoonotic infections [41].

### Human-microbe interactions

At least three transitions of the human-microbe interaction occurred after about 10,000 yBP [40]. The first, called the Neolithic revolution, led to domestication of plants and animals, and to the passage from nomadic to sedentary life in the Middle East. Many microbes originating from husbanded animals would have had contact with humans and failed to prosper. Some of them, however, would have survived causing infections such as influenza, smallpox, cholera, and tuberculosis.

The second transition refers to empowered Eurasian civilizations that came in economic and military contact 1,500 to 3,000 yBP, swapping their germ pools with often disastrous results [42]. These included the Justian Plague of AD 542 that devastated Constantinople and massive epidemics in China at about the same time.

The third transition was consequence of the European imperialism from the XV^th^ century onwards. It led to the trans-oceanic spread of a big repertoire of infections brought by the conquerors [42]. This did not only happen in the Americas but also as a consequence of the European exploration of Asia and Australia and of the slave-trade from Africa to various parts of the world. This favored the spread of numerous parasitic diseases with specific strains according to geographic area.

The main question asked herein is where, in this intricate sequence of events, should the origin of syphilis be placed.

### The paleopathological data

As a first attempt to answer this question, we plotted all available pre-Columbian evidences of bony treponematoses on a world map. This meant we accepted the risk of different methods and criteria for the establishment of differential diagnosis reported in the previously published studies. This also meant that we acknowledge the possibility that the following interpretations on the osteological analysis alone might be flawed.

**Figure 4:**
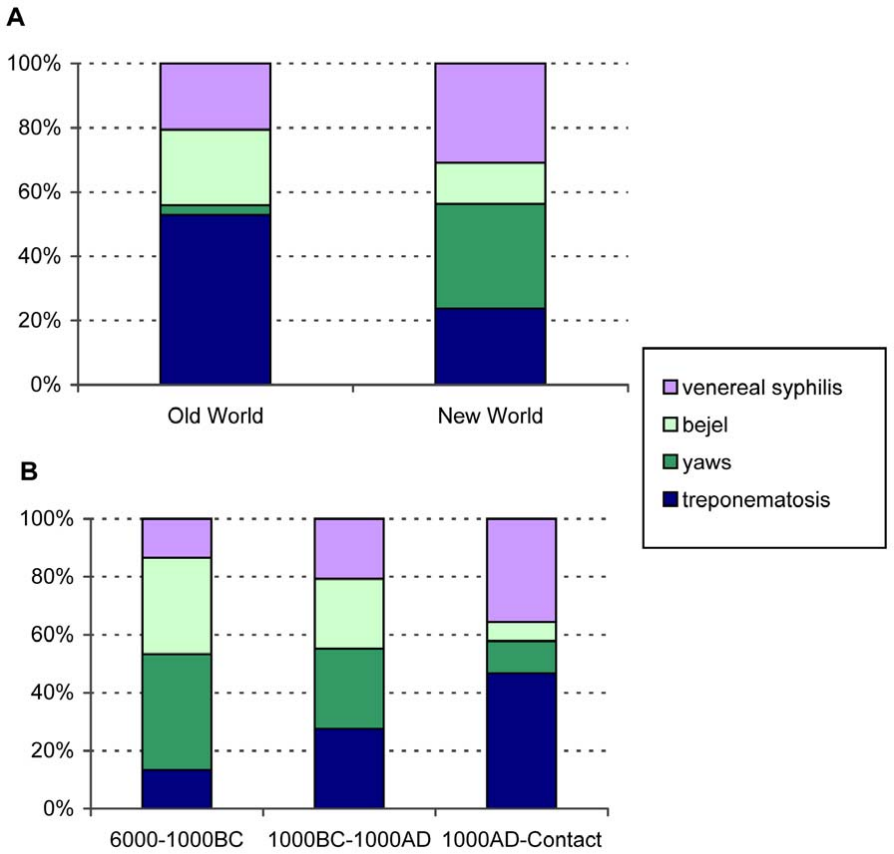
Geographical and temporal distribution of treponematoses. A) Percentage of Old World (N = 33) and New World (N = 56) cases of treponematoses, excluding those with uncertain diagnosis or date. B) Percentage of the same cases taken together and distributed according to the corresponding time intervals: 6,000–1,000 BC (N = 15), 1,000 BC–AD 1,000 (N = 29) and AD 1,000-Contact (N = 45). These distributions are based on Table 1.

Our literature review produced a total of 128 pre-Columbian treponematoses cases worldwide (Figure 3, Table 1, and Table S1). Excluding cases with uncertain diagnoses or age and those with “probable” diagnoses (and thus considering only original reports with clear diagnoses and dating), there are almost twice as many treponematoses cases in the New (N = 56), than in the Old World (N = 33). This is not surprising if considering that systematic studies regarding these pathologies have been more frequently undertaken in North America than elsewhere. If excluding from these cases all those with the broad diagnosis of treponematosis, there are significantly more cases of bejel in the Old (Old World: 8/16, New World: 7/42; p = 0.0172) and significantly more cases of yaws in the New World (Old World: 1/16; New World: 18/42; p = 0.0108), while venereal syphilis is evenly distributed (Old World: 7/16; New World: 17/42; p = 0.8210), as seen in Figure 4A. This probably reflects climatic conditions that favor the manifestation of bejel in the more arid Old World and yaws in the often hot and humid areas of the New World. However, this could also be due to a bias caused by a higher number of publications by scientists (e.g. Rothschild) whose strong belief is that yaws was the only treponematosis present in the New World at the time. On the other hand, this also means that the reports of syphilis in Europe and Russia long before the XVI^th^ century contradict the assumption that syphilis was the “*New World's revenge upon Columbus and his crew*” [43]. Thus, considering only osseous evidences of treponematosis, it appears that venereal syphilis afflicted humans thousands of years ago in the New as well as in the Old World (Figures 3 and 4a, Table 1).

**Table 1:** New and Old World distribution of pre-Columbian treponematoses cases, as evidenced by paleopathological research.

	Total cases	New World cases	Old World cases	Probable cases	Uncertain diagnosis or age	Total
**6,000–1,000 BC**						
Treponematosis	2	2	0	0	0	**2**
Yaws	6	5	1	1	1	**8**
Bejel	5	2	3	0	1	**6**
Syphilis	2	1	1	1	0	**3**
Total	**15**	**10**	**5**	**2**	**2**	**19**
**1,000 BC–1,000 AD**						
Treponematosis	8	5	3	1	1	**10**
Yaws	8	8	0	3	2	**13**
Bejel	7	3	4	0	0	**7**
Syphilis	6	5	1	4	1	**11**
Total	**29**	**21**	**8**	**8**	**4**	**41**
**1,000 AD- Contact**						
Treponematosis	21	7	14	2	5	**28**
Yaws	5	5	0	4	1	**10**
Bejel	3	2	1	1	0	**4**
Syphilis	16	11	5	3	7	**26**
Total	**45**	**25**	**20**	**10**	**13**	**69**
**Grand Total**	**89**	**56**	**33**	**20**	**19**	**128**

For references please refer to Table S1. Probable or uncertain cases were excluded from the quantification of treponematoses cases excavated in the New and Old World.

The number of established differential diagnoses (or clear cases of venereal, syphilis, bejel, yaws and treponematosis in the peer reviewed articles) increases substantially from the more ancient (6000–1000 BC, N = 15) to the two more recent periods (1000 BC–AD 1000, N = 29; AD 1000-contact, N = 45), as seen in Table 1. This is due to the higher number and the better state of conservation of more recent skeletal collections compared to those exhumed from more ancient sites. The progressive decrease of yaws (6/13; 8/21, 5/24) and bejel cases (5/13; 7/21; 3/24), although suggested in Figure 4B, is not statistically significant. However, there is a huge and significant rise especially in venereal syphilis cases from 1000 BC to the time of Contact (6/21 to 16/24; p = 0.0169), as already noted for North America in the book “The Myth of Syphilis” [11]. While this may be attributed to an increase of the venereal form of treponematosis, especially in the New World (5/16 to 11/18; p = 0.1005), the Old World syphilis cases cannot be ignored. So, considering solely these palaeopathological reports, the Columbian hypothesis seems not to explain the origin of syphilis.

On the other hand, it also seems clear that treponemal diseases in general were well established in pre-Columbian times all over the world. The oldest dates we are aware of refer to bejel in Sudan 15,000 yBP, yaws in Florida some 7,900 yBP, and treponematosis in Peru more than 8,000 yBP (Figure 3, Table 1, Table S1). Nevertheless, the microevolutionary sequence proposed by Hackett in 1963, although a good working hypothesis, does also not account for all osseous evidences of the treponematoses evaluated [14]. According to Hackett (1963) [14], bejel is seen as typical of arid environments, but there are evidences of bejel in the area of former Yugoslavia [44] and one in today's Canada [45]. Additionally, Hackett proposed that bejel arose from yaws about 9,000 yBP, but the 15,000 years old bejel case from Sudan [46] pushes this transition back some six thousand years. These data suggest that the scheme proposed by Hackett does not match the evidences anymore.

Finally, the Unitarian hypothesis predicts that each social group had the treponemal disease compatible with the prevailing environmental and cultural conditions. However, no clear climatic, ecological or cultural pattern emerges when analyzing Figure 3, Table 1 and Table S1.

### Difficulties in differential diagnosis

It is important to remark that there is controversy regarding the criteria for the establishment of differential diagnosis of treponematoses in osteological collections [11],[47],[48]. So far, there are two main schools of paleopathological interpretation of skeletal lesions representative of treponemal diseases: one very specific [49] and another extremely broad [50]. Thus, the conclusions based solely on paleopathology reports have to be taken very cautiously. This also means that there is an urge to standardize diagnostic criteria, if the history of treponematoses is to be unraveled. Until this “gold standard” is established, tested and accepted (vis-à-vis the fragmentary nature of the archaeological record and the osteological paradox [51]), a reassessment of all claims of treponemal diseases registered until the present would be unfruitful. Furthermore, despite the enormous efforts, there still remains controversy on the differential diagnosis of treponematoses also in living humans, due to partially overlapping symptoms and modes of transmission - that seem to be defined by opportunity rather than biology [20].

### Changes in virulence

Another important issue refers to the changing virulence of syphilis over time. First mentioned as evil pocks in the Edict of the Holy Roman Emperor Maximilian, the devastating epidemics in the late XV^th^ and early XVI^th^ century were assumed by some scientists and historians to have been venereal syphilis. Although it seems clear that it was caused by a sexually transmitted disease, the evidence that it was syphilis is less certain [11]. In any case, it rapidly turned to be a very aggressive disease, killing within a few years [14]. Today, however, untreated patients with syphilis can survive for decades. This strongly suggests that this disease, also called the great imitator or the great impostor, changes with time. This would mean either the bacteria lost virulence during time or that syphilis killed off the most susceptible population right in the beginning of its devastating existence.

### Combining paleopathological and molecular data

As discussed above analyzing pre-Columbian cases of treponematoses based on osseous remains renders no clear picture on the origin and spread of these infectious diseases. This suggests the necessity of employing other methodologies. Here we test if using osseous evidences to calibrate phylogenetic analyses of the treponemes yields plausible results. In order to explore the efficacy of this new integrative method we investigate the three scenarios discussed below.

### Treponematosis in *Homo erectus*?

Using 1.6 million years as the date of the first evidence of treponematosis in the genus *Homo*, as that reported for *H. erectus* [30], we found a mean rate of 6.35×10^−10^ substitutions/site/year for the *T. pallidum* group (Table 2). This is about ten times slower in comparison to other bacteria (*E. coli*: 4.5–5.0×10^−9^, *Buchnera* 8.2×10^−9^ subs/site/year [34]), and even slower than the rate observed in humans (Eukaryotes and humans: 2.5 to 0.4×10^−9^ [52]). Since in bacteria generation time is much smaller and effective population size is many times bigger than in humans, and as genetic diversity increases proportionally with generation time and effective population size [34], this slow evolutionary rate seems very unlikely for the *T. pallidum* group.

**Table 2:** Test of hypothesis A.

	Substitution rate	MRCA of *T. pallidum* subsp. *pertenue*	MRCA of *T. pallidum* subsp. *pallidum*
Mean^a^	6,35E-10	641,000	1,220,000
95% HPD lower	8,44E-11	51,000	87,600
95% HPD upper	1,44E-09	1,750,000	3,180,000

Most Recent Common Ancestor and Substitution rates estimated for the different subspecies of *Treponema pallidum* constraining the group root to 1.6 million years BP according to the fossil evidence of treponematosis in *H. erectus* [30].

a years before present.

Furthermore, this analysis shows a temporal overlap of the most recent common ancestor of *T. pallidum* subsp. *pertenue* and *T. pallidum* subsp. *pallidum* from 175,000 to 87,600 yBP (Table 2). This suggests that both these treponemes existed since at least about 88,000 years ago, meaning that yaws as well as venereal syphilis should have accompanied successful human migrations since that time.

In case the lesions found in the *H. erectus* fossil really can be attributed to treponematosis and if our phylogenetic analysis is correct, we expect to encounter evidences of treponemal diseases, including a true syphilitic Eve, in bones as ancient as 88,000 years ago, not only in our own species, but also in our cousin species, such as in the Neanderthals. A fact that turns this possible is that related treponemes have been found to affect other than the human species, including African monkeys [53] and rabbits [54]. Thus, it is feasible that the treponeme species affecting humans was originally a zoonosis [55], as already suggested by Hackett in 1963.

Despite the observation of treponematosis in *H. erectus* 1.6 million years ago [30], no other sign of treponemal diseases was found until now, older than about 15,000 years ago (Table S1).

On the other hand, there are some evidences of pre-Columbian venereal syphilis outside the New World, *i.e.* in Europe, Russia, and Japan (Figures 3, 4A, Table 1 and Table S1). In France, this evidence is strong since there is an infant with signs of congenital syphilis buried during the Late Roman Empire, amongst other cases such as the fetus from Costebelle [10],[15],[56] (Table S1). Other pre-Columbian Old-World cases of congenital syphilis were found in the ancient Greek colony Metaponto (dated to 580–250 BC) in today's Italy [57] and in XIII^th^ century Turkey [23]. That is, all continents, except Africa and Oceania show pre-Columbian evidences of either venereal or congenital syphilis.

That Africa shows no cases as yet of ancient syphilis is easily explained by the scarce paleopathology studies undertaken there until now. However, in Oceania many such studies have been carried out, so this argument is not valid.

Although there is only one case of treponematosis plotted for Australia on Figure 3, we are aware of more such cases on this continent prior to the arrival of the Europeans in the XVIII^th^ century. This includes not only endemic forms found in the arid parts of this continent, but also some cases that could match a diagnosis of venereal syphilis [58]. Since the exact number and location of skeletons with treponematosis buried prior to the arrival of the Europeans was unavailable, Australia is underrepresented in our analyses.

Considering the unlikely evolutionary rate found using the evidence of treponematosis in *H. erectus* to calibrate phylogenetic analysis and the absence of osseous treponemal disease older than 15,000 BP anywhere in the world, it is far from probable that the treponematoses, as we know them today arose more than a million years ago. The treponematosis diagnosed in *H. erectus* [30] can thus have been caused not by the treponeme strains analyzed herein, but by ancestral forms still unknown.

### Is genetic variation in *T. pallidum* subsp. *pallidum* just 500 years old?

As a next step, we tested the hypothesis that *T. pallidum* subsp. *pallidum* emerged in Europe from less virulent strains caught in the New World by Columbus' crew about 450–550 yBP [14],[19],[59]. Assuming this hypothesis, the most recent common ancestor of the whole group of *T. pallidum*, was found to be dated to 3,900 yBP (Table 3). This would mean either that the treponematoses originated in the New World about 4,000 years ago, or that they emerged at this time in the Old World and were then brought to the New World.

**Table 3:** Test of hypothesis B.

	Substitution rate	MRCA all	MRCA of *T. pallidum* subsp. *pertenue*
Mean^a^	1,41E-06	1,818	248
95% HPD lower	2,96E-07	477	21
95% HPD upper	2,98E-06	3,903	649

Most Recent Common Ancestor and Substitution rates estimated for the different subspecies of *Treponema pallidum* constraining the TMRCA of *T. pallidum* subsp. *pallidum* to Columbus' return to Europe (450–550 yBP [19]).

a years before present.

In the first case, treponematoses would have spread from the New World to the rest of the globe mainly during the Great Voyages. However, this interpretation would not explain the existence of treponematoses dated to before 4,000 yBP in the Old World, such as the cases from Sudan, Egypt, India and Russia (Figure 3, Table S1).

In the case treponematoses emerged in the Old World by 4,000 yBP and were brought by more recent migrational waves to the New World, this would explain the pre-Columbian treponematoses finds in Europe and Asia (Figures 3 and 4, Table S1), but does not allow treponematoses in New World bones older than 4,000, as for example the case of yaws registered in bones as old as 7,900 yBP excavated in Florida [39] or the treponematosis from a circa 5,000 yBP riverine shell mound in Brazil [22].

Additionally, the results of this analysis imply that *T. pallidum* evolved exceptionally fast, with a substitution rate of 1.41×10^−6^ substitutions/site/year (95% HPD of 2.96×10^−7^ to 2.98×10^−6^). If assuming the lower HPD as true, *T. pallidum* evolved at a rate at least 100 times faster than *E. coli* (4.5–5.0×10^−9^ subs/site/year [34]), but similar to that found in *Helicobacter pylori* (6.2×10^−7^ to 9.2×10^−7^ [60]). Since this last organism lacks a DNA repair system [61] one would expect that *T. pallidum*, which has a repair system [37], would show a rate slower than that of *H. pylori*. Thus, a treponeme substitution rate in the order of 10^−6^ to 10^−7^ would be unlikely. Moreover, despite limited, comparative studies with *T. pallidum* sequences have shown a small amount of variability in the treponeme genome, suggesting a high conservation degree between the various subspecies [62],[63]. A high conservation degree has also been observed among sequences of *Tpr* genes of *T. pallidum* subsp. *pallidum* strains collected at different times (in the early 1900s and in the final years of the 20th century) [18]. Since the *Tpr* are the most variable genes of the *T. pallidum* genome, this result corroborates the idea that the substitution rate in *T. pallidum* is possibly not high. Consequently, this points to a slow substitution rate in the treponemes as a whole. Therefore, the scenario that venereal syphilis emerged as recently as 500 years ago seems utterly unlikely, be it in the New or the Old World.

### Did venereal syphilis emerge in the Americas between 16,500 and 5,000 years ago?

Using the root constrains of 16,500 yBP, when the first humans are believed to have entered the Americas [31], and 5,000 yBP (the oldest osseous evidence of venereal syphilis in the New World [32]), we found that the upper 95% HPD of the most recent common ancestor for the treponeme group is 77,400 yBP (Table 4). This result does not allow yaws in *H. erectus*, but could explain the claims of pre-Columbian treponematoses in *H. sapiens* across the world. Therefore, according to the phylogenetic approach used herein, our data do not contradict that syphilis might have emerged in the New World [19]. However, based on our paleopathology survey, we cannot exclude that venereal syphilis emerged in the Old World.

**Table 4:** Test of hypothesis C.

	Substitution rate	MRCA all	MRCA of *T. pallidum* subsp. *pertenue*
Mean^a^	8,82E-08	32,100	4,407
95% HPD lower	1,25E-08	5,858	385
95% HPD upper	2,01E-07	77,400	12,500

Most Recent Common Ancestor and Substitution rates estimated for the different subspecies of *Treponema pallidum* constraining the root of *T. pallidum* subsp. *pallidum* to the entrance of humans into the New World (16,500 yBP [31]) and the oldest evidence of venereal syphilis in the Americas (circa 5,000 yBP [32]).

a years before present.

Additionally, the estimated evolutionary rate was found to be 8.82×10^−8^ substitutions/site/year (Table 4). This rate is plausible if compared to other bacterial rates. But this result has to be taken carefully, since the evolutionary rate varies between organisms, as consequence of differences in life traits [34]. On the other hand, there is evidence that variation in generation time does not affect evolutionary rates in some bacterial lineages [64]. Consequently, our estimations may be reliable.

Therefore, to independently test this evolutionary rate, we used the oldest and most strongly supported osseous evidences (bejel in Sudan 15,000 yBP [46] and congenital syphilis in France 1,600 yBP [10],[15],[56],[65]) to constrain the TMRCA of the whole group of *T. pallidum* and the TMRCA of *T. pallidum* subsp. *pallidum*. The resulting rate was 4.09×10^−8^ (varying from 6.82×10^−8^ to 2.62×10^−9^ substitutions/site/year – data not shown). Despite the difference, these rates partly overlap, suggesting that the range estimated testing hypothesis c) is likely.

### What do the whole genome comparisons tell us?

In addition to the hypotheses tested and discussed above, we also compared the rate of substitutions between the two whole genome strains of *T. pallidum* subsp. *pallidum* reported by Matejkova et al. (2008) [38]. If considering 5,300 yBP at Indian Knoll in the United States [59] and 5,000 yBP in Colombia [32] as the oldest and “safest” osseous evidences of venereal syphilis in the World (Figure 3 and Table S1), the resulting rate is 2.1×10^−8^ substitutions/site/year. When, however, considering the oldest osseous evidence of congenital syphilis as being that reported for Costebelle in France circa 1,600 yBP [65] and for Metaponto in Italy about 2,400 yBP [57], these values correspond to 9.25×10^−8^ and 6.16×10^−8^ substitutions/site/year respectively (data not shown). Finally, if considering that venereal syphilis emerged only some 500 years ago from the less virulent strains brought to Europe by Columbus, we found 2.92×10^−7^ substitutions/site/year. Whereas the first three rates vary in the same range as that reported for *E. coli* and *Buchnera* [34], the rate of 2.92×10^−7^ is far too fast. This, again, argues against a very recent origin of venereal syphilis, in contrast to Harper et al. (2008) and others [19].

### Limitations and strengths

Our study harbors the back draws that limit every paleopathological as well as phylogenetic study. More specifically, the limits of this work are as follows: First, although the worldwide map on pre-Columbian treponemal bones contains as much data as we could collect, totalizing 128 cases, many reports on ancient treponematoses could not be included in our study. Second, since the diagnostic criteria differ between the reports on treponematoses in ancient bones, one cannot exclude that reassessing all possible cases with standardized methods would lead to different results. Third, since there is little sequence variation within and between the *Treponema* strains available, our highest probability density (HPD) values are large, reflecting the uncertainty inherent in almost all molecular date estimates. Forth, the absence of a suitable molecular clock rate for the *T. pallidum* group also limits the possibilities of estimating more accurate dates for the emergence of specific clades of treponemes. Therefore, we can not exclude that the evolutionary rate of *T. pallidum* lies outside the range observed for other bacteria. Last but not least, we could not advance knowledge of the place of origin of syphilis. Thus, our results have to be taken cautiously.

Despite these limitations, we believe that the major strength of this work is the cross-fertilization of two so distinct areas as paleopathology and molecular phylogenetics. This novel approach enabled us to study phylogenetics of the treponemes with the advantage of knowing where the characteristic bony lesions appeared first in the history of our ancestors, thus significantly enhancing analytical quality.

### Recommendations

Despite the decades of enormous research efforts to describe and classify the different treponemes and treponematoses, both in active infections and in archaeological collections, some important hallmarks have to be reached to unravel the origin and spread of venereal syphilis. These include the establishment of diagnostic criteria to clearly distinguish between the three treponemal diseases that affect the skeleton, as well as selecting candidate cases in understudied osteological collections. After the establishment of this “gold standard” a reassessment of the well contextualized osteological collections worldwide has to be undertaken using a population approach. On the other hand, more phylogenetic studies of the whole treponeme group, including the saprophytic, zoonotic, and commensal species, as also those affecting primates and other mammals have the potential to aid in this effort. The more frequent use of new and complementary techniques, such as immunohistochemical studies of mummies [29] can also significantly enhance our comprehension on the origin and spread of this challenging disease.

### Conclusions

The use of evidences from both, paleopathology and molecular phylogenetics, provided a broad and integrative approach to the study on the origin of treponematoses. If our analysis using treponematosis in *H. erectus* is correct, we expect to encounter very ancient evidences of treponemal diseases, including a true syphilitic Eve. Since the oldest evidence reaches only 15,000 yBP and given that the evolutionary rate found is far to low compared to other bacteria and also to humans, treponematosis, as we know it today, seems very unlikely in a 1.6 million years old *H. erectus*. On the other hand, when constraining the TMRCA of *T. pallidum* subsp. *pallidum* to Columbus' return to Europe some 500 years ago, the estimated evolutionary rate was considerably higher than expected for bacteria, and does not explain the majority of the osseous record. Thus, both these hypotheses seem improbable. Alternatively, the estimated evolutionary rate reaches values comparable to other bacteria, when we fix the TMRCA to between 16,500 and 5,000 yBP. Consequently, venereal syphilis seems to have emerged around this time. This would allow the claim of venereal syphilis in pre-Columbian times throughout the world.

Most importantly, rather than trying to pinpoint the emergence time of venereal syphilis, we used treponematoses to test our transdisciplinary approach aimed at reconstructing the evolutionary history of different human infectious diseases. As described above, we independently explored three scenarios. The retrieved results corroborate the currently most accepted views on the origin of syphilis. This opens the possibility to use this approach to shed light on other diseases that afflict humankind since immemorial times.

## Supporting Information

Table S1 Referenced list of archaeological sites containing pre-Columbian evidences of treponematosis, yaws, bejel and venereal and congenital syphilis used to construct Figure 3.
(0.17 MB XLS)

Table S2 Genbank acession numbers for all the sequences used in the phylogenetic analysis.
(0.03 MB XLS)

Alternative Language Abstract S1 Translation of the abstract into German by SE.
(0.02 MB DOC)

Alternative Language Abstract S2 Translation of the abstract into Portuguese by FLM.
(0.03 MB DOC)
