# Supplementary material for: Syphilis at the Crossroad of Phylogenetics and Paleopathology
Source: PLoS Negl Trop Dis. 2010 Jan 5;4(1):e575. doi: 10.1371/journal.pntd.0000575 (PMC2793018; doi:10.1371/journal.pntd.0000575)
Supplement: Alternative Language Abstract S1 — Translation of the abstract into German by SE. (0.02 MB DOC) [file pntd.0000575.s003.doc]

**Abstract**Der Ursprung der Syphilis ist umstritten. Verschiedene Methoden und Forschungsansätze wurden herangezogen, um die Entwicklung dieser faszinierenden Pathologie zu untersuchen. Hier haben wir dafür einen neuen integrativen Ansatz benutzt und getested, bei dem Paläopathologie und molekulare Phylogenetik kombiniert werden. Das Ziel ist verschiedene Szenarien über den Ursprung der Syphilis und anderer Krankheiten die auch von Treponemen verursacht werden (Treponematosen), zu unteruchen. Zunächst haben wir Ort, Datierung und Diagnose aller zugänglichen Berichte über prä-Kolumbianische Treponematosen am menschlichen Skelett auf einer weltweiten Karte versammelt. Dann wählten wir die ältesten Fälle und kallibrierten damit den Zeitpunkt des letzten gemeinsamen Vorfahren verschieder Subspezien der Bakterie Treponema (*T. pallidum* subsp*. pallidum, T. pallidum* subsp*. endemicum* und *T. pallidum* subsp. *pertenue*) in einer phylogenetischen Analyse, die auf 21 genetischen Regionen dieser Bakterienstämme basiert. Dann haben wir die Evolutionsrate der Treponema-Stämme errechnet, um die folgenden Hypothesen zu testen: a) Treponematosen haben die menschliche Entwicklung seit *Homo erectus* begleitet; b) Syphilis entwickelte sich aus weniger virulenten Neu-Welt Stämmen vor circa 500 Jahren, und c) Syphilis ist vor 16.500 bis 5.000 Jahren in Nord-, Mittel- und/oder Süd-Amerika entstanden. Zwei der resultierenden Evolutionsraten sind unplausible und ausserdem nicht mit den paläopathologischen Befunden zu vereinen: Die Treponematosen, wie wir sie heute kennen, sind nicht mit *H. erectus* aufgetaucht; Syphilis ist auch nicht erst vor fünhundert Jahren entstanden. Wenn wir jedoch die Datierung der Erstbesiedelung Amerikas (vor rund 16.500 Jahren) und die des ältesten Syphilisfundes (vor etwa 5.000 Jahren) zum Kalibrieren heranziehen, ist Hypothese c) nicht gänzlich zu falsifizieren: Syphilis scheint in dieser Zeitspanne entstanden zu sein, da die errechnete Evolutionsrate mit der anderer Bakterien übereinstimmt. Allerdings, wenn die Ansprüche der präkolumbischen Syphilisfälle außerhalb Amerikas berücksichtigt werden, bleibt der Herkunftsort ungeklärt. Das Bestreben Paläopathologie und Phylogenese zu vereinen erwies sich als fruchtbarer und vielversprechender Ansatz um auch die Entstehungsgeschichte anderer Infektionskrankheiten zu untersuchen.
