# Supplementary material for: Syphilis at the Crossroad of Phylogenetics and Paleopathology
Source: PLoS Negl Trop Dis. 2010 Jan 5;4(1):e575. doi: 10.1371/journal.pntd.0000575 (PMC2793018; doi:10.1371/journal.pntd.0000575)
Supplement: Alternative Language Abstract S2 — Translation of the abstract into Portuguese by FLM. (0.03 MB DOC) [file pntd.0000575.s004.doc]

**Resumo**

A origem da sífilis é ainda hoje controversa, e diferentes linhas de pesquisas exploram sua fascinante história. Neste trabalho empregamos um método integrativo combinando dados paleopatológicos e moleculares. No intuito de testar esta metodologia, diferentes hipóteses sobre a origem da sífilis e de outras treponematoses humanas foram avaliadas. Inicialmente, foi construído um mapa-múndi contendo todas as evidências paleopatológicas acessíveis de treponematoses pré-Colombianas. Em seguida, foram selecionadas as evidências mais antigas para fixar a data de origem do ancestral comum mais recente de *Treponema pallidum* subsp. *pallidum*, *T. pallidum* subsp. *endemicum* e *T. pallidum* subsp. *pertenue.* Estas análises foram realizadas utilizando seqüências de 21 regiões do genoma de diferentes linhagens de *T. pallidum*. Foram estimadas as taxas evolutivas para as três hipóteses testadas: a) se treponematoses existem desde *Homo erectus*; se a sífilis venérea b) emergiu recentemente de linhagens menos virulentas vindas do Novo Mundo, cerca de 500 anos atrás, e c) emergiu na América entre 16.500 e 5.000 anos atrás. Duas das taxas evolutivas resultantes foram improváveis e não explicam todas as evidências ósseas existentes: as treponematoses, tal como as conhecemos hoje, não emergiram com *H. erectus*, nem a sífilis venérea apareceu há apenas cinco séculos atrás. Considerando 16.500 anos atrás como data da entrada do homem nas Américas, e 5.000 anos atrás, como a evidência mais antiga e provável de sífilis venérea no mundo, não podemos rejeitar totalmente a hipótese c). Uma vez que a taxa evolutiva resultante é compatível com taxas observadas em outras bactérias, confirmamos que a sífilis venérea pode ter surgido neste período. Entretanto, se as evidências pré-colombianas de sífilis venérea fora das Américas forem levadas em consideração, o local de origem permanece indefinido. Finalmente, o esforço em agregar dados paleopatológicos e análises filogenéticas demonstrou ser uma abordagem frutífera e promissora para estudar outras doenças infecciosas.
